# Social determinants of delivery mode in Jiangsu, China

**DOI:** 10.1186/s12884-019-2639-2

**Published:** 2019-12-05

**Authors:** Hong Fan, Hai Gu, Hua You, Xinpeng Xu, Yun Kou, Nichao Yang

**Affiliations:** 1 Center for Health Policy and Management Research, Nanjing University, 22 Hankou Road, Nanjing, People’s Republic of China; 2 Department of Social Medicine and Health Education, School of Public Health, Nanjing Medical University, 101 Longmian Road, Nanjing, People’s Republic of China

**Keywords:** Social determinants, Delivery mode, Jiangsu, China

## Abstract

**Background:**

Less evidence exists regarding the association of social determinants and delivery mode in Jiangsu, and if the trend is influenced by the type of residence. This study aims to identify the significant social determinants of delivery mode, and also to compare the main differences in delivery mode between urban and rural areas.

**Methods:**

We used data from the cross-sectional National Health Service Surveys conducted in Jiangsu Province in 2013. For the purposes of this study, information from women (15–64 years old) who had experienced childbirth the last 5 years were examined, and a total of 1365 participants were selected as research subjects.

**Results:**

Participants using vaginal delivery mode and cesarean delivery mode were found in 616 (45.1%) and 751(54.9%) participants, respectively. The proportion of women using cesarean delivery was 53.5% in rural area and 58.2% in urban area. Meanwhile, our results showed that women in middle Jiangsu were more likely to use cesarean delivery, and cesarean delivery is more prevalent among richer women. We also find that the more use of prenatal care visit, the more use of cesarean delivery.

**Conclusions:**

This study validated the relationship between social determinants and the mode of delivery in Jiangsu province. Social determinants are contextual factors, which may vary by region and additional work is needed to fully understand these relationships globally. Further studies are needed to elucidate mechanisms and pathways across various populations, and these social determinants should be incorporated into future multi-level interventions designed to decrease the cesarean delivery rate.

## Background

According to the findings from National Health services in China, the cesarean delivery (CD) rate rose from 6.1 to 41.0% between 1998 and 2013. The CD rates in big cities were much higher than that in rural areas. However, the rates increased dramatically both in rural and urban areas during the period. CD rates were associated with local economic conditions: the higher the local GDP per capita, the higher CD rate [1]. Jiangsu, an economically highly-developed province in China, is one of regions with high CD rates in China. CD rate in Jiangsu was 54.8% reported by the 2013 National Health Services Survey. International and domestic research concluded that unnecessary CD raised the risk of potential adverse health consequences both for maternal and infants. Moreover, it also caused a heavy economic burden on individual, family and local government. It was estimated that the expenditure on medical care associated with unnecessary CD was approximately US$ 2.32 billion globally [2]. China has made remarkable achievements in improving maternal and child health, but the dramatic rise of unnecessary CD rates, especially in China’s big cities, should be arouse our attention.

Social determinants in medicine area can be defined as the social and economic conditions that influence health status, including socio-demographic characteristics and socio-economic determinants [3]. Numerous social determinants may contribute to this increasing unnecessary CD rates in China. Firstly, fear of pain and safety were the most frequently expressed reasons for preferring CD. Pregnant women who often worry about their fitness or sexual function after giving birth naturally would also prefer for their baby to be delivered by CD. Secondly, due to that Chinese astrology has been prevailing in China for thousand years, many people believe that the birthday and birth time determines people’s destiny, which is a common reason why some Chinese people would pick a lucky date and time to give birth by CD [4]. And last but not least, high rates of unmarried pregnancy and later pregnancy, the pressure from family planning policy, and the economic incentives in CD medical practices, are also the common underlying reasons for the unnecessary CD.

However, less evidence exists regarding the association of social determinants and delivery mode in Jiangsu, and if the trend is influenced by the type of residence. It is significant to compare the delivery mode in Jiangsu and to identify the social determinants influencing it.

The purpose of this study was to identify the significant social determinants of delivery mode. We also analyzed urban and rural areas separately because of differences in socio-demographic and socio-economic factors [5]. This study is unique due to the combination of multiple factors within the same population and on the use of a sample size large enough to allow analysis while controlling for important covariates.

## Methods

### Data source and sampling

We collected data from the cross-sectional National Health Service Surveys conducted in Jiangsu Province in 2013.Jiangsu is located in southeast China, with a population of 79.98 million in the end of 2016 and an area of 107,200 km^2^. Regions in Jiangsu Province are customarily separated into three geographic regions: south Jiangsu, middle Jiangsu and north Jiangsu. These three geographic regions are collectively composed of 13 prefecture-level city cities and 96 counties [6]. Areas belonging to the same geographic region share similar social-economic status. Generally, the south Jiangsu has the best economic performance, followed by the middle Jiangsu and the worst performance in the north Jiangsu. In this study, the county is defined as the primary sampling unit.

A multi-stage, stratified, cluster, random sampling method was employed in this survey. Firstly, eighteen cities and counties were randomly selected according to the socio-economic status. Secondly, five districts or townships were selected within each city or county, respectively. Thirdly, two villages were sampled within each district or township, and lastly a random sample of households was selected from each village. A total of 12,600 households were finally selected.

All the interviews were conducted by trained investigators using a structured questionnaire which included the general information on the maternal and infants’ characteristics (see Additional file 1). The traced back-period was 5 years for the survey and collected recorded information on the last live birth [5]. All the participants provided the informed consent. Ethical approval including the consent procedure was obtained from the Research Ethics Committee of Nanjing University, Nanjing, China.

### Measures

For the purposes of this study, we only examined the information from women (15–64 years old) who had experienced childbirth the last 5 years, and we analyzed only a limited number of the comprehensive list of socio-demographic and socio-economic indicators included in the questionnaires because many were correlated with each other and because we wished to focus on the social determinants [5]. The variables selected were: maternal age at delivery, type of residence, region, household income per capital, mother’s education attainment, access to health insurance, children ever born, number of prenatal care, self-reported delivery mode and delivery institution.

To simplify interpretation of the odds ratio (OR), participants’ household income per capita (HIPC) measures were divided into four classes based on the urban residents’ disposable income per capita (URDIPC) or on the rural residents’ net income per capita (RRNIPC) of their respective city/town from the corresponding year [income level 1 (HIPC< 50% URDIPC/ RRNIPC), income level 2 (50% URDIPC/ RRNIPC≤HIPC<URDIPC/ RRNIPC),income level 3 (URDIPC/ RRNIPC ≤HIPC< 200% URDIPC/ RRNIPC) and income level 4 (HIPC≥200% URDIPC/ RRNIPC)]. There are currently five medical insurance programs in Jiangsu, which are Urban Employee Basic Medical Insurance (UEBMI), Urban Resident Basic Medical Insurance (URBMI), and New Cooperative Medical Scheme (NCMS), Urban and Rural Residents Cooperative Medical Insurance (URCMI) and Commercial Medical Insurance (CMI) [7] .

### Data analysis

All data were processed using the program SPSS 22.0 (SPSS, Inc., Chicago, IL, USA) and Epidata 3.1. We used QQ plots and Kolmogorov–Smirnov to test the normality of distribution Multivariate logistic regression analysis was performed to identify social determinants associated with the delivery mode. In the logistic regression model, we adopted the Enter Method to achieve a final model. The standard for the variable inclusion was based on SLE = 0.10, and the exclusion standard was SLS = 0.15. Odds Ratios (OR) and 95% Confidence Intervals (CI) were calculated, and *P* values< 0.05 were considered statistically significant.

## Results

A total of 1367participants (mean age 29 ± 5.7 years, maternal age 13–58 years) were included in the present study. Participants using vaginal delivery (VD) mode and cesarean delivery (CD) mode were found in 616(45.1%) and 751(54.9%) participants, respectively. Mean number of prenatal visits was around 7.3(±3.5) for women using VD and 7.8(±4.9) for women using CD during pregnancy.

Except for the overall information, Table 1 also shows the socio-demographic and socio-economic characteristics of the women stratified by the delivery mode in urban and rural areas separately. The proportion of women using CD was 53.5% in rural area and 58.2% in urban area. Except for the maternal age, the significant socio-demographic and socio-economic characteristics differences were found between rural and urban women undergoing CD.

**Table 1:** Baseline characteristics by mode of delivery

	Rural		Urban		All		*P*
	VD	CD	VD	CD	VD	CD	
Total	440(46.5)	506(53.5)	175(41.8)	244(58.2)	615(45.1)	750 (54.9)	
Maternal age at delivery (yrs)							
< 35	380(86.4)	445(87.9)	161(92.0)	224(91.8)	541(88.0)	669(89.2)	0.475*
≥ 35	60(13.6)	61(12.1)	14(8.0)	20(8.2)	74(12.0)	81(10.8)	
Type of residence							
Rural	–	–	–	–	440(71.5)	506(67.5)	
Urban	–	–	–	–	175(28.5)	244(32.5)	
Region							
South Jiangsu	209(47.5)	249(49.2)	107(61.1)	119(48.8)	316(51.4)	368(49.1)	0.002*
Middle Jiangsu	77(17.5)	116(22.9)	12(6.9)	46(18.9)	89(14.5)	162(21.6)	
North Jiangsu	154(35.0)	141(27.9)	56(32.0)	79(32.4)	210(34.1)	220(29.3)	
Household income per capital							
Income level 1	104(23.6)	92(18.2)	24(13.7)	32(13.1)	128(20.8)	124(16.5)	0.005*
Income level 2	226(51.4)	228(45.1)	60(34.3)	87(35.7)	286(46.5)	315(42.0)	
Income level 3	95(21.6)	150(29.6)	66(37.7)	87(35.7)	161(26.2)	237(31.6)	
Income level 4	15(3.4)	36(7.1)	25(14.3)	38(15.6)	40(6.5)	74(9.9)	
Mother’s education							
No formal education	17(3.9)	14(2.8)	1(0.6)	0(0.0)	18(2.9)	14(1.9)	0.236*
Primary	54(12.3)	51(10.1)	3(1.7)	2(0.8)	57(9.3)	53(7.1)	
Secondary	291(66.1)	359(70.9)	65(37.1)	83(34.0)	356(57.9)	442(58.9)	
College and above	78(17.7)	82(16.2)	106(60.6)	159(65.2)	184(29.9)	241(32.1)	
Health insurance							
None	11(2.5)	18(3.6)	17(9.7)	20(8.2)	28(4.6)	38(5.1)	0.095**
UEBMI	62(14.1)	86(17.0)	122(69.7)	172(70.5)	184(29.9)	258(34.4)	
URBMI	30(6.8)	26(5.1)	30(17.1)	41(16.8)	60(9.8)	67(8.9)	
NCMS	244(55.5)	301(59.5)	6(3.4)	9(3.7)	250(40.7)	310(41.3)	
URCMI	90(20.5)	73(14.4)	0(0.0)	0(0.0)	90(14.6)	73(9.7)	
CMI	3(0.7)	2(0.4)	0(0.0)	2(0.8)	3(0.5)	4(0.5)	
Children ever born							
0	278(63.2)	320(63.2)	146(83.4)	213(87.3)	424(68.9)	533(71.1)	0.394*
≥ 1	162(36.8)	186(36.8)	29(16.6)	31(12.7)	191(31.1)	217(28.9)	
Number of Prenatal care							
< 5	104(23.6)	87(17.2)	16(9.1)	16(6.6)	120(19.5)	103(13.7)	0.004*
≥ 5	336(76.4)	419(82.8)	159(90.9)	228(93.4)	495(80.5)	647(86.3)	
Delivery institution							
Primary institution	168(38.2)	192(37.9)	13(7.4)	25(10.2)	181(29.4)	217(28.9)	0.841*
Secondary and above	272(61.8)	314(62.1)	162(92.6)	219(89.8)	434(70.6)	533(71.1)	

*VD* Vaginal Delivery, *CD* Cesarean Delivery, *UEBMI* Urban Employee Basic Medical Insurance, *URBMI* Urban Resident Basic Medical Insurance, *URCMI* Urban and Rural Residents Cooperative Medical Insurance, *NCMS* New Cooperative Medical Scheme, *CMI* Commercial Medical Insurance

Data shown is number (percentage) for all variables

*P value is based on Chi-square test which was used to determine whether there is a significant difference on related variable between rural and urban women undergoing CD

**P value is based on Fishers exact test which was used to determine whether there is a significant difference on related variable between rural and urban women undergoing CD

Significant differences were observed for region, income level and number of prenatal care (Table 2). Participants in middle Jiangsu (OR, 1.485; 95% CI, 1.077–2.046) reported a higher use of CD than south. Women from income level 4 households were more likely to use CD (OR, 1.661; 95% CI, 1.013—2.724) than the women from income level 1. The rate of CD increased in tandem with the number of prenatal visits. Women who made five and above prenatal visits were 1.418 times (95% CI, 1.038–1.939) more likely to use CD than the participants who made less than 5 prenatal visits.

**Table 2:** Multivariate logistic regression for the association of social determinants with the mode of delivery

Variables	Odds Ratio(95%CI)	*P* value
Maternal age at delivery (ref = 34 or below)	1.050(0.714–1.545)	0.803
Type of residence (ref = Rural)	1.099(0.794–1.520)	0.568
Region (ref = South Jiangsu)		
Middle Jiangsu	1.485(1.078–2.047)	0.016
North Jiangsu	0.957(0.721–1.270)	0.758
Household income per capital (ref = Income level 1)		
Income level 2	1.051(0.771–1.433)	0.755
Income level 3	1.353(0.952–1.923)	0.092
Income level 4	1.658(1.011–2.720)	0.045
Mother’s education (ref = No formal education)		
Primary	1.080(0.481–2.427)	0.852
Secondary	1.317(0.624–2.780)	0.469
College and above	1.155(0.517–2.579)	0.725
Health insurance (ref = None)		
UEBMI	0.956(0.553–1.652)	0.872
URBMI	0.858(0.466–1.581)	0.624
NCMS	0.980(0.563–1.706)	0.944
URCMI	0.632(0.338–1.183)	0.152
CMI	0.872(0.178–4.280)	0.866
Children ever born (ref = 0)	1.082(0.816–1.434)	0.586
Number of Prenatal care (ref = 5 or below)	1.413(1.034–1.933)	0.030
Delivery institution (ref = Primary institution)	1.048(0.804–1.367)	0.727

*UEBMI* Urban Employee Basic Medical Insurance, *URBMI* Urban Resident Basic Medical Insurance, *URCMI* Urban and Rural Residents Cooperative Medical Insurance, *NCMS* New Cooperative Medical Scheme, *CMI* Commercial Medical Insurance

### Social determinants of delivery mode in rural area

The results of the multivariate logistic regression analysis assessing the social determinants of the mode of delivery in rural area are presented in Table 3. Compared with women from income level 1 households, the likelihood of undergoing CD is significantly high for women from income level 3(OR,1.622;95% CI,1.061—2.480) and income level 4(OR, 2.587; 95% CI,1.279—5.230). Women with URCMI reported a lower use of CD at around 57% (OR, 0.430; 95% CI, 0.183—1.010) than women who did not have any formal health insurance.

**Table 3:** Multivariate logistic regression for the association of social determinants with the mode of delivery in rural area

Variables	Odds Ratio(95%CI)	*P* value
Maternal age at delivery (ref = 34 or below)	0.936(0.600–1.460)	0.770
Region (ref = South Jiangsu)		
Middle Jiangsu	1.121(0.765–1.643)	0.558
North Jiangsu	0.762(0.523–1.111)	0.158
Household income per capital (ref = Income level 1)		
Income level 2	1.034(0.721–1.483)	0.855
Income level 3	1.618(1.058–2.473)	0.026
Income level 4	2.582(1.277–5.221)	0.008
Mother’s education (ref = No formal education)		
Primary	0.976(0.428–2.228)	0.954
Secondary	1.130(0.526–2.427)	0.753
College and above	0.799(0.339–1.881)	0.607
Health insurance (ref = None)		
UEBMI	0.775(0.327–1.833)	0.562
URBMI	0.579(0.222–1.506)	0.262
NCMS	0.802(0.363–1.770)	0.585
URCMI	0.427(0.182–1.004)	0.051
CMI	0.407(0.057–2.894)	0.369
Children ever born (ref = 0)	1.233(0.895–1.700)	0.200
Number of Prenatal care (ref = 5 or below)	1.381(0.972–1.963)	0.071
Delivery institution (ref = Primary institution)	1.174(0.878–1.570)	0.279

*UEBMI* Urban Employee Basic Medical Insurance, *URBMI* Urban Resident Basic Medical Insurance, *URCMI* Urban and Rural Residents Cooperative Medical Insurance, *NCMS* New Cooperative Medical Scheme, *CMI* Commercial Medical Insurance

### Social determinants of delivery mode in urban area

Table 4 shows the results of the multivariate logistic regression analysis assessing the social determinants of the mode of delivery in urban area. Women in middle Jiangsu (OR, 3.801; 95% CI, 1.885–7.665) reported a higher use of CD than women in south Jiangsu. Contrastingly, there were no statistical differences in the rate of CD with regard to the maternal age at delivery, household income per capital, mother’s education attainment, access to health insurance, children ever born, number of prenatal care, and delivery institution.

**Table 4:** Multivariate logistic regression for the association of social determinants with the mode of delivery in urban area

Variables	Odds Ratio(95%CI)	*P* value
Maternal age at delivery (ref = 34 and below)	1.491(0.661–3.362)	0.336
Region (ref = South)		
Middle	3.801(1.885–7.665)	0.000
North	1.294(0.822–2.035)	0.265
Household income per capital (ref = Income level 1)		
Income level 2	1.282(0.665–2.471)	0.458
Income level 3	0.940(0.480–1.841)	0.857
Income level 4	0.981(0.440–2.187)	0.963
Mother’s education (ref = < College)		
College and above	1.305(0.820–2.078)	0.262
Health insurance (ref = YES)		
NO	0.982(0.476–2.025)	0.961
Children ever born (ref = 0)	0.702(0.371–1.327)	0.276
Number of Prenatal care (ref = 5 or below)	1.441(0.676–3.074)	0.344
Delivery institution (ref = Primary institution)	0.557(0.268–1.159)	0.117

## Discussion

In the present study, we find that urban women have higher CD rates than rural women, which is similar to the findings in other developing countries. For example, research from Pakistan and 26 countries in Southern Asia or Sub-Saharan Africa both declare the same fact that a rising trend of CD presented in urban areas [8–10]. Firstly, the increasing availability and accessibility of CD in urban areas may contribute to the widely utilization [11]. Secondly, women in urban areas usually have a better socio-economic status, for example, from better educational background, with higher employment rates, and with better economic level, which can influence the affordability of CD [2]. However, we noticed that the proportion of women using CD in rural areas was also very high (53.5%). A possible explanation is that, with recent economic development in rural areas, there has been a great change in attitudes towards CD among rural women. Moreover, people have been able to get imbursement for the medical cost of CD with the implementation of NCMS. All of the above factors contribute to the high rates of CD in rural areas. Unnecessary CD raised potential risks both for mothers and infants and also resulted in unnecessary expenditure on medical care. High rates of CD, especially in rural areas, have become a potentially alarming phenomenon worthy of attention.

We also analyzed the association of social determinants and the mode of delivery. According to statistical analysis, the results showed that women in middle Jiangsu were more likely to use CD. We are, however, limited in ability to explain this finding, as, to our knowledge, there is very limited research on spatial variation. One likely explanation is that, the middle Jiangsu in Jiangsu (Yangzhou, Taizhou and Nantong), especially in poorer areas, have a tendency to use CD, which is partly due to the local cultural influence [12]. In addition, compared to the number of health providers in south Jiangsu, there are staffing and physical limitations of the public facilities in maternal care in middle Jiangsu, resulting in the higher use of CD services which is an efficient way to move patients through the systems [13].

CD is more prevalent among richer women in our findings, which is in accordance with the results of studies conducted in Asia and Africa [8, 9]. The possible reason for such difference is that richer women may be delivering in more expensive or highly rated institutions, which may be more likely to perform CD for economic incentives considering that the rich women can afford the extra cost associated with delivery mode. Moreover, the rich women are more likely to be fear about the painful experience caused by natural delivery which may prompt them to consider CD [9, 14]. It is implied that developing means of improving the childbirth experience may relieve maternal anxiety aiming at reducing unnecessary CD [15].

We also find that the more use of prenatal care visit, the more use of CD. The prenatal care is important for both mothers and babies, and viewed as an essential measure for a normal pregnancy. It is likely that many underlying complications are found by prenatal care, and which increase the possibilities of undergoing CD. And the women who made more prenatal care visits may tend to adopt CD [16]. This finding has significant public health implication: if necessary measures can be taken to prevent the incidence of complications during the prenatal period, it may dramatically reduce the frequency of unnecessary CD.

Our data suggest that the socio-economic status of residence region is a more important determinant of delivery mode than the women’s individual socio-economic characteristics in urban area [17]. We found that the CD rate was almost four times higher among women in middle Jiangsu than in south Jiangsu, irrespective of individual variations. Hence, it makes sense to identify these non-individual social determinants and to analyze how they influence the delivery mode. Supply-side factors may be a more important determinant of CD than individual factors in urban area [18].

There are two limitations that should be noted. Firstly, the study design was cross-sectional and in consequence the ability to adequately address issues such as causality may have been impaired. This issue could potentially be resolved by the collection of data longitudinally or by using path analysis and structured equation modeling on cross-sectional data as a means of better understand the underlying relationships. Secondly, the possible existence of confounding factors (such as maternal disease and access to health care) ought to be taken into account in future studies.

## Conclusions

This study validated the relationship between social determinants and the mode of delivery in Jiangsu province. Social determinants are contextual factors which may vary by region. Accordingly, these social determinants ought to be incorporated into future multi-level interventions designed to decrease the CD rate, with further studies required in order to properly elucidate mechanisms and pathways across various populations.

## Supplementary information

**Additional file 1.** Questionnaire for women aged 15–64.

## Data Availability

The datasets used and/or analyzed during the current study are available from the corresponding author on reasonable request.

## Abbreviations

CD: Cesarean Delivery
CMI: Commercial Medical Insurance
GDP: Gross Domestic Product
HIPC: Household Income per capita
NCMS: New Cooperative Medical Scheme
OR: Odds Ratio
RRNIPC: Rural Residents’ Net Income per capita
UEBMI: Urban Employee Basic Medical Insurance
URCMI: Urban and Rural Residents Cooperative Medical Insurance
URDIPC: Urban Residents’ Disposable Income per capita
VD: Vaginal Delivery
