# Supplementary material for: Social determinants of delivery mode in Jiangsu, China
Source: BMC Pregnancy Childbirth. 2019 Dec 5;19:473. doi: 10.1186/s12884-019-2639-2 (PMC6894495; doi:10.1186/s12884-019-2639-2)
Supplement: Supplementary file 1 — Additional file 1. Questionnaire for women aged 15–64. [file 12884_2019_2639_MOESM1_ESM.docx]

**Questionnaire for women aged 15-64**

**Women born between 16 August 1949 and 15 August 1998**

| Identification code numbers | | 01 | 02 | 03 | 04 | 05 | 06 |
| --- | --- | --- | --- | --- | --- | --- | --- |
|  | Have you had any gynecological examination in the last 12 months?  ⑴Yes ⑵No |  |  |  |  |  |  |
|  | Have you had a cervical smear in the last 12 months? ⑴Yes ⑵No |  |  |  |  |  |  |
|  | Have you had a breast exam in the last 12 months? ⑴Yes ⑵No |  |  |  |  |  |  |
|  | (In rural area) when you wish to be served by a female doctor in a township health center, were you able to get it?  (1)Yes ⑵No (3) Did not think about it (4)Not sure |  |  |  |  |  |  |
|  | (In rural area) Has your husband worked in other place for a long time?  ⑴Yes ⑵No |  |  |  |  |  |  |
|  | How many times have you been pregnant? |  |  |  |  |  |  |
|  | How many children have you had? |  |  |  |  |  |  |
|  | Date of your last delivery: (year) (in 4 digits, e.g. 1998) |  |  |  |  |  |  |
|  | (month) (in 2 digits, e.g. 07) |  |  |  |  |  |  |
| The following questions are for women who have given birth after 15 Aug,2008 | | | | | | | |
|  | Gender of your last child: (1) Male (2)Female |  |  |  |  |  |  |
|  | How many prenatal tests did you have when you were having your last child? |  |  |  |  |  |  |
| Identification code numbers | | 01 | 02 | 03 | 04 | 05 | 06 |
|  | When you were doing prenatal examination, have you had blood test?  ⑴Yes ⑵No |  |  |  |  |  |  |
|  | Have you measured your blood pressure? ⑴Yes ⑵No |  |  |  |  |  |  |
|  | Have you had your urine checked? ⑴Yes ⑵No |  |  |  |  |  |  |
|  | Have you had an ultrasound test? ⑴Yes ⑵No |  |  |  |  |  |  |
|  | How was the child born: (1) Natural birth (2)Cesarean section |  |  |  |  |  |  |
|  | For cesarean section, who mainly suggested this?  (1)Self-inquire ⑵Doctor’s advice (3)Others’ advices |  |  |  |  |  |  |
|  | Where did you give birth?  (1) County hospital and above (2) Health center for maternal women and children  (3) Health care institutions in villages and towns  (4) Community health service center  (5) Health service station (6) At home (7 )Other place |  |  |  |  |  |  |
|  | If you delivered at home, who was the midwife:  (1) Doctors in town areas or bigger areas; (2) Doctors in villages  (3) Professional midwife (4)Non-professional midwife  (5)Family member (6)Other people |  |  |  |  |  |  |
|  | How heavy was the baby born? |  |  |  |  |  |  |
|  | How much was the total cost of delivery? |  |  |  |  |  |  |
|  | How much did you pay by yourself? (excluding reimbursement and expenses in personal medical accounts) |  |  |  |  |  |  |
|  | How many times (times) did you receive postpartum visits within 42 days after delivery? |  |  |  |  |  |  |
